# Pervasiveness of Biological Impacts of Artificial Light at Night

**DOI:** 10.1093/icb/icab145

**Published:** 2021-06-25

**Authors:** Kevin J Gaston, Simone Ackermann, Jonathan Bennie, Daniel T C Cox, Benjamin B Phillips, Alejandro Sánchez de Miguel, Dirk Sanders

**Affiliations:** Environment & Sustainability Institute, University of Exeter, Penryn, Cornwall TR10 9FE, UK; Environment & Sustainability Institute, University of Exeter, Penryn, Cornwall TR10 9FE, UK; Environment & Sustainability Institute, University of Exeter, Penryn, Cornwall TR10 9FE, UK; Environment & Sustainability Institute, University of Exeter, Penryn, Cornwall TR10 9FE, UK; Environment & Sustainability Institute, University of Exeter, Penryn, Cornwall TR10 9FE, UK; Environment & Sustainability Institute, University of Exeter, Penryn, Cornwall TR10 9FE, UK; Environment & Sustainability Institute, University of Exeter, Penryn, Cornwall TR10 9FE, UK

## Abstract

Artificial light at night (ALAN) and its associated biological impacts have regularly been characterized as predominantly urban issues. Although far from trivial, this would imply that these impacts only affect ecosystems that are already heavily modified by humans and are relatively limited in their spatial extent, at least as compared with some key anthropogenic pressures on the environment that attract much more scientific and public attention, such as climate change or plastic pollution. However, there are a number of reasons to believe that ALAN and its impacts are more pervasive, and therefore need to be viewed from a broader geographic perspective rather than an essentially urban one. Here we address, in turn, 11 key issues when considering the degree of spatial pervasiveness of the biological impacts of ALAN. First, the global extent of ALAN is likely itself commonly underestimated, as a consequence of limitations of available remote sensing data sources and how these are processed. Second and third, more isolated (rural) and mobile (e.g., vehicle headlight) sources of ALAN may have both very widespread and important biological influences. Fourth and fifth, the occurrence and impacts of ALAN in marine systems and other remote settings, need much greater consideration. Sixth, seventh, and eighth, there is growing evidence for important biological impacts of ALAN at low light levels, from skyglow, and over long distances (because of the altitudes from which it may be viewed by some organisms), all of which would increase the areas over which impacts are occurring. Ninth and tenth, ALAN may exert indirect biological effects that may further expand these areas, because it has a landscape ecology (modifying movement and dispersal and so hence with effects beyond the direct extent of ALAN), and because ALAN interacts with other anthropogenic pressures on the environment. Finally, ALAN is not stable, but increasing rapidly in global extent, and shifting toward wavelengths of light that often have greater biological impacts.

## Introduction

There has long been recognition that the introduction of artificial light into the nighttime environment has biological impacts. Early observations particularly highlighted the delayed retention of leaves on trees close to, and the attraction of insects and birds toward, outdoor light sources (e.g., Allen 1880; Gastman 1886; Matzke 1936; Schroeder 1945; Cochran and Graber 1958; Verheijen 1960). Nonetheless, it is only recently that artificial light at night (ALAN) has been regarded as a significant anthropogenic environmental pressure (Longcore and Rich 2004; Rich and Longcore 2006; Hölker et al. 2010; Gaston et al. 2014; Gaston 2018; Owens et al. 2020). This development has particularly been spurred by two things. First, satellite observations of the Earth at night have highlighted the widespread geographic occurrence of direct ALAN emissions, detected as the vertically emitted or reflected component (Sullivan 1989; Román et al. 2018; Levin et al. 2020), and this has been further emphasized by modeling of the extent of skyglow (indirect ALAN; artificial brightening of the night sky that results predominantly from upwardly emitted artificial light being scattered in the atmosphere by water, dust, and gas molecules; Cinzano et al. 2001; Falchi et al. 2016).

Second, a rapidly growing body of studies has documented many ways in which the disruption of natural light regimes by ALAN has biological impacts. These include at the levels of the individual (e.g., physiology, behavior; Da Silva et al. 2015; Brüning et al. 2018; Grubisic et al. 2019), the population or species (e.g., abundance, distribution, reproduction, mortality, dispersal; Stone et al. 2012; Dominoni et al. 2013; Gaston and Bennie 2014; Davies et al. 2017; Rodríguez et al. 2017), the community (e.g., species composition and richness, trophic structure; Sanders and Gaston 2018), through to the ecosystem (e.g., pollination, seed dispersal; Lewanzik and Voigt 2014; Knop et al. 2017). Impacts have been documented in all environmental realms (marine, freshwater, terrestrial), in a wide array of habitat types, and across microbes, plants, fungi, and animals (Gaston et al. 2013; Bennie et al. 2016; Sanders et al. 2021). The mechanisms by which these effects occur are increasingly well understood (Gaston et al. 2013; Dominoni 2015; Falcon et al. 2020).

This said, how pervasive or systemic the biological impact of ALAN actually is, and in this sense how comparable to other anthropogenic pressures on the environment, remains rather unclear. On the one hand, ALAN continues to be characterized widely as essentially an urban issue, and to be discussed most commonly in the context of towns and cities. While its effects would then be tightly linked to where most people occur, this would actually suggest a relatively limited spatial extent compared to many other anthropogenic pressures (e.g., climate change, ocean acidification, plastic pollution). Estimates of global urban coverage are highly variable—in large part dependent on definitions of urban land cover, and the spatial resolution and accuracy of the data used—but consensus seems to be that this is less than 1% of the land surface (e.g., Potere et al. 2009; Schneider et al. 2009; Li et al. 2017). Some national and regional urban coverages can be several-fold larger, but are almost invariably still quite limited (excepting some small highly developed nations and regions; Zhou et al. 2015). Of course, these zones of influence are markedly increased by the inclusion of skyglow, but this has recently been estimated still “only” to occur over less than a quarter of global land area (Falchi et al. 2016).

On the other hand, natural light regimes are the strongest and most predictable environmental fluctuations that organisms typically experience and play a fundamental role in biology (Bradshaw and Holzapfel 2010; Gaston et al. 2017). They can be exquisitely sensitive to variation in these regimes, through diel, lunar and seasonal cycles. Thus, for example, (i) at low latitudes where annual variation in day length is less than an hour, some plant species are still using such changes as biological cues (e.g., Borchert and Rivera 2001; Rivera and Borchert 2001); (ii) in the high Arctic polar night zooplankton undertake diel vertical migration response to diel variation in solar radiance despite the sun never rising above the horizon (Ludvigsen et al. 2018); and (iii) nocturnal insects can see color, fly, navigate, maneuver at fine scales and land at known targets at very low nighttime light levels (Sponberg et al. 2015; Warrant 2017). This would suggest that altering natural light regimes even mildly could have potent biological effects.

In this paper, we highlight a series of factors that suggest that the biological effects of ALAN may indeed be spatially much more pervasive than is often understood. We focus foremost on factors that have in the main received limited research attention, often because this is challenging to undertake. We consider, first estimations of the extent of ALAN. We then turn to a series of ways in which ALAN may be more widespread than an “urban” characterization would imply. We address, in turn, isolated sources, mobile sources, marine systems, remote sources, light levels, skyglow, elevation, landscape ecology, interactions with other factors. Finally, we consider what future changes might portend. We end with some concluding remarks. Throughout, our intent is not to provide a comprehensive systematic review, but rather to explore and link key issues and provide some examples.

## Estimations of the extent of ALAN

Remote sensing data, especially those derived from satellite-borne sensors, have played a pivotal role in documenting the extent, composition and dynamics of ALAN (Levin et al. 2020). Indeed, although other platforms are becoming available (Colomb et al. 2003; Li et al. 2019; Zhang et al. 2019), most understanding of these issues has arisen from data obtained from the Defense Meteorological Satellite Program Operational Linescan System (DMSP-OLS) and, more recently, the Visible Infrared Imaging Radiation Suite (VIIRS) on the Suomi National Polar-Orbiting Partnership satellite. While they have been invaluable, these sources have important limitations. In determining the extent of direct emissions of ALAN these include that

- the spatial resolution of data acquisition remains quite coarse (DMSP-OLS—2.7 km; VIIRS—740 m), making them better suited to detecting emissions from collections of, rather than individual, lights;
- variation in the angle of data acquisition means that emissions from some sources of artificial lighting are more apparent than others, with emissions close to the nadir, and those from well shielded and more horizontally projected sources, being less detectable (combining data obtained at multiple different times and thus different angles can reduce this issue);
- emissions that are shielded by natural vegetation (e.g., under tree canopies, as may occur in some rural, typically tropical, communities) will not be well detected;
- data are consistently acquired at particular local times of day when artificial light may not necessarily be at its peak and which means that the inevitable diel variation is not captured (DMSP-OLS—20.00; VIIRS—01.30);
- sensors are panchromatic, and insensitive to emissions in the blue part of the spectrum which are particularly associated with the broad “white” light-emitting diode (LED) technology that is increasingly being used in outdoor lighting (Bierman 2012). This can result in substantial underestimation (e.g., 50%) of the overall intensity of ALAN emissions;
- sensors are sensitive to emissions in the infrared, and thus to those from volcanoes and fires, as well as high pressure sodium (HPS) lamps; and
- some ALAN emissions will be removed when processing data to reduce contributions of lighting from non-ALAN sources, such as airglow, the aurora and fires. This is particularly true of ALAN emissions that appear to vary on short timescales, either because they genuinely do so (e.g., because of limited or erratic availability of power, or because they are mobile), or because they appear to do so as a consequence of variation in the angles of data acquisition at different times.

Many of these limitations are greatly exacerbated by the need to obtain satellite data in the absence of substantial cloud cover, conditions that occur infrequently in some regions and times of year. Many of the limitations have variously been overcome with the use of other sensor platforms (e.g., balloons, drones, manned aircraft, the International Space Station), but these have thus far been extremely limited in the ground/sea coverage that has been achieved and in the frequency of repeat data acquisition for the same areas (Levin et al. 2020).

**Fig. 1:**
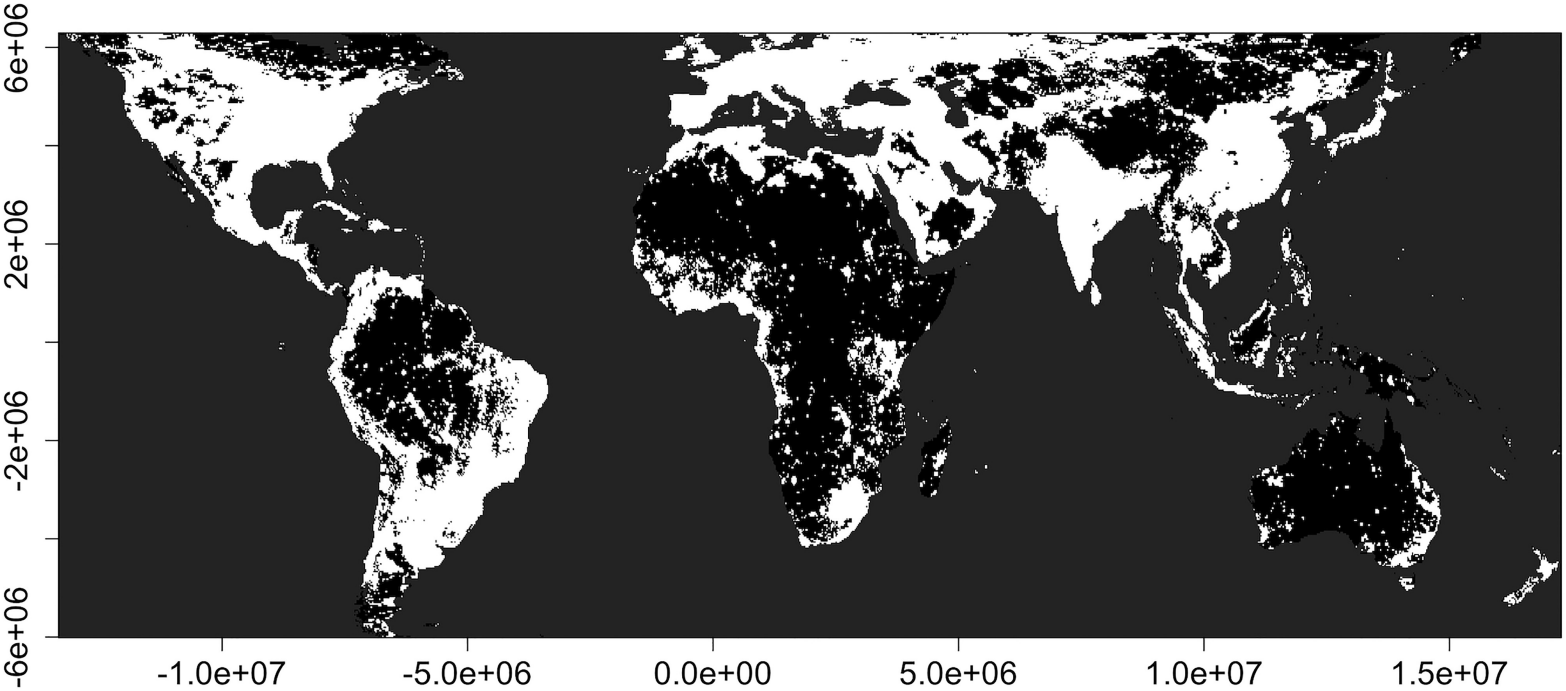
Spatial variation in artificial light at night over the global land surface. The layer gives an estimated combined extent of direct emissions and skyglow between 59°N and 55°S at 1.61 × 1.21 km resolution in Behrmann equal area projection (see Cox et al. 2020a for a full description of methods). In brief, the layer gives VIIRS day/night band (DNB) values corrected for albedo and skyglow. To avoid the confounding effects of the aurora and permanent snow and ice, we excluded pixels above 59°N and 55°S. Once albedo had been removed, DNB airglow was detected over the majority of the land surface. Airglow varies with latitude and therefore to provide the best possible mask while minimizing the obscuring of true direct ALAN emissions, for each 200 km latitudinal band we calculated the median DNB value and converted all values below the median to 0 (mean across latitudinal bands 0.098; min 0.015, max 0.202). To incorporate skyglow we used values for artificial brightness from the New World Atlas of Artificial Sky Brightness (Falchi et al. 2016). Following Falchi et al. (2016), we set artificial brightness values below 0.00,174 mcd/m^2^ to 0, because these were considered indistinguishable from a pristine night sky. To map the extent of both forms of ALAN, values of skyglow were added to values of direct ALAN emissions before converting all values greater than 0 to 1. and thereby creating a binary layer of where ALAN is present (white) or absent (black).

**Fig. 2:**
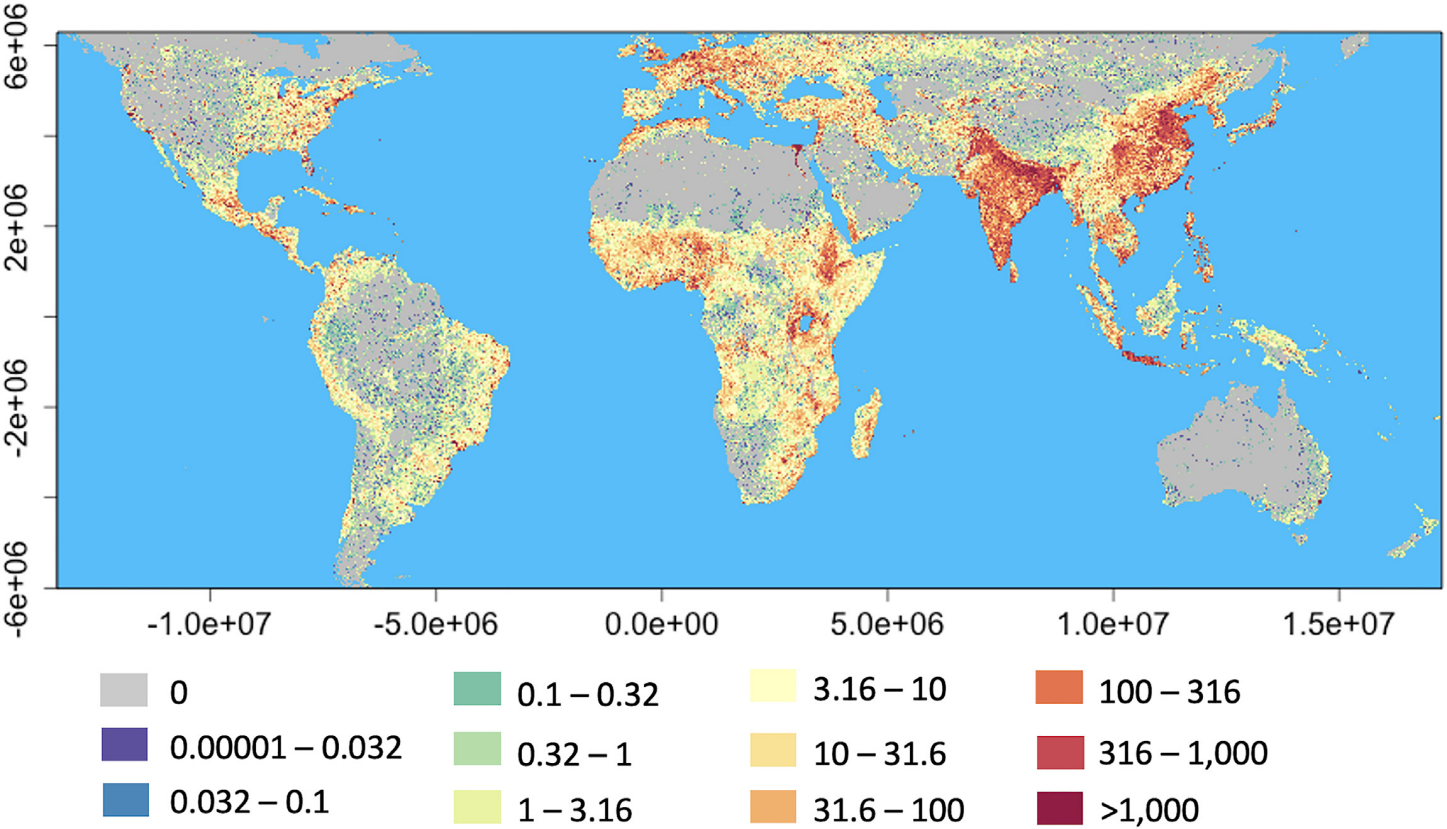
Spatial variation in human population density. The layer was created by adjusting the raw LandScan 2016 population count layer for area and projecting to Behrmann equal area (EASE-Grid 2.0: EPSG:6933; Brodzik et al. 2012) to give population density per km^2^ at a resolution of 1.61 × 2.12 km^2^. The color banding is log10 at intervals of 0.25.

Direct light can be observed at an almost indefinite distance from the source (subject to the curvature of the earth and atmospheric scattering), but shading, geometric dilution and the attenuation of light in the atmosphere mean that direct illuminance declines with distance from the source and can vary by orders of magnitude over short distances. Any estimate of the global area affected by ALAN emissions is therefore necessarily dependent on the spatial resolution used to measure it and the intensity threshold used. Processing data from VIIRS to estimate the global coverage of both direct ALAN emissions and skyglow, at a spatial resolution of 1.61 km × 2.12 km, ALAN extends over 49.5% of the land surface between 59°N and 55°S (Fig. 1; this is where confounding effects of albedo, airglow, the aurora and permanent snow and ice on imagery are relatively small). By way of comparison, when sampled at the same resolution a recent attempt to map ambient human densities (averaged over 24 h) reveals that between the same latitudinal limits people and ALAN co-occur over 39.4% of the global land surface, but that people occur over 63% of that surface (Fig. 2). While there are doubtless areas in which people are present but producing little or no ALAN emissions outside of buildings, the combination of these estimates suggests that the extent of ALAN may indeed be markedly underestimated by satellite data in isolation due to low detection thresholds. Such satellite data, and models based upon them, have frequently been used to assess the extent of the biological impacts of ALAN (e.g., Bennie et al. 2014a; Duffy et al. 2015; Gaston et al. 2015; Correa-Cano et al. 2018; Koen et al. 2018; Garrett et al. 2020). These may thus prove to be significant underestimates.

## Isolated sources

Much of the difference between the extent of ALAN as estimated from nighttime satellite data and as inferred from the distribution of people will arise from the occurrence of lighting at low densities outside of towns and cities, and away from foci of industrial activity (e.g., airports, ports, power stations, mining sites) and major transport arteries (e.g., motorways). Indeed, in understanding the biological impacts of ALAN it is likely to be important to distinguish between two situations. The first is that in which these emissions arise from multiple sources and essentially contribute to most local organisms living in a much altered “light environment”; this is linked to various attempts to determine what contribution different kinds of sources (from streetlights, housing, businesses, advertisement hoardings, etc.) make to this environment (Kuechly et al. 2012; Kyba et al. 2021). The second situation is that in which the effect of ALAN on organisms is driven by their responses to individual light sources. As evidenced by a recent formal meta-analysis (Sanders et al. 2021), to date studies of the biological impacts of ALAN have tended to focus foremost on the former circumstance. This has typically involved observations in the vicinity of streetlights or subjecting organisms to experimental treatments intended to simulate such light conditions. This is, in effect, also a focus on the impacts of downward emissions from light sources, and that is often how these are narratively and figuratively predominantly conceptualized.

Considering the isolated light sources that predominate in more rural areas focuses attention on an issue that is of less importance in urban areas, namely how far emissions are spatially propagated. For true diffuse point sources, the illuminance of a receiving plane decreases with the square of the distance from the light source (the inverse square law):

$$\begin{equation*}
E = k\left( {I/{r^2}} \right)
\end{equation*}$$

where *E* is illuminance, *I* is the luminous intensity of the source, *r* is the distance, and *k* a constant (dependent on the units used; Schreuder 2010). Hence, illuminance at or close to the horizontal from isolated inadequately shielded streetlights and other such sources will tend to decline quite quickly. When a source is small in relation to the distance this equation also works as an approximation for non-point sources, and large sources can usefully be treated as multiple small ones. For “bundled” sources, where emissions are shaped into a parallel beam by a mirror or lens, the inverse square law underestimates the level of illuminance at a given distance with, all else being equal, the luminous intensity remaining roughly constant with distance. Indeed, for such sources, aside from intervening obstacles (vegetation, buildings, topography), the distance over which they can be detected is principally determined by the curvature of the earth. Most external lighting sources (such as streetlights) fall closer to the inverse-square law than a focused, bundled source, but some spotlights, floodlights, vehicle headlights and some modern LED lighting fixtures emit more parallel beams of light and so luminance will decline with distance at a much slower rate.

The distances over which different organisms respond to isolated artificial light sources remain surprisingly poorly understood. Most attention in this regard has been paid to insects, particularly in connection with understanding the areas over which light traps are effectively sampling. For moths, estimates of this distance vary greatly but suggest that attraction is limited—within generally a few meters (Baker and Sadovy 1978); generally <30 m for 50% of individuals (Beck and Linsenmair 2006); generally <10 m, but up to 80 m (Truxa and Fiedler 2012); generally <10 m, but up to 50 m (van Grunsven et al. 2014); or up to at least 50 m (Plaut 1971). For midges, this distance is generally 2–4 m (Venter et al. 2012), but up to a maximum of 15.5 m (Kirkeby et al. 2013). This suggests that for the majority of individuals these distances are low, although given that in the main these studies are based on mark-recapture over short periods, the potential that some individuals may travel much greater distances remains.

The other group for which there is some information on distances over which individuals respond to artificial light sources is seabirds (a group that can experience high mortality as a result of such attraction; Rodríguez et al. 2017). In the main this information is indirect, being based on the separation between colonies and the sources of lighting (e.g., Imber 1975; Rodrigues et al. 2012; Rodríguez et al. 2014; Syopsz et al. 2018). However, one study using GPS data-loggers to track the maiden and second flights of Cory's shearwater *Calonectris diomedea* fledglings from nest-burrows found that these were grounded by artificial lights at distances up to 16 km (Rodríguez et al. 2015a). Another found that fledglings of three species of petrels were grounded by artificial lights at a mean distance of ∼5 km and up to >20 km from their colonies (Rodríguez et al. 2015b).

Similar kinds of measurements do not seem to have been conducted for other groups of organisms, and thus the range of attraction of artificial light sources is essentially unknown. Moreover, equivalent experiments testing repulsion effects of isolated lights have not been done.

In addition to attraction or repulsion, isolated artificial lights in landscapes could have other effects, including through visual confusion or distraction. For example, fireflies and glow worms may find it harder to detect light signals made by other individuals (e.g., Owens and Lewis 2018; Desouhant et al. 2019; Lewis et al. 2020). Attraction of isolated lights can also have second-order effects, for example through attraction of predators (e.g., spiders, frogs, bats) to aggregations of prey that have been directly attracted (Canário et al. 2012; Minnaar et al. 2015; Rodríguez et al. 2021).

## Mobile sources

Attention to ALAN predominantly focuses on static sources of emissions (e.g., streetlights, building lights). Many mobile, and therefore often temporally sporadic, sources (e.g., from road vehicles, rail vehicles, shipping) will not be detected by remote sensing because they are projected predominantly in a horizontal plane, and indeed, if they are detected may be removed in the processing of data to reduce the influence of fires (natural and human-caused) and gas flares.

**Fig. 3:**
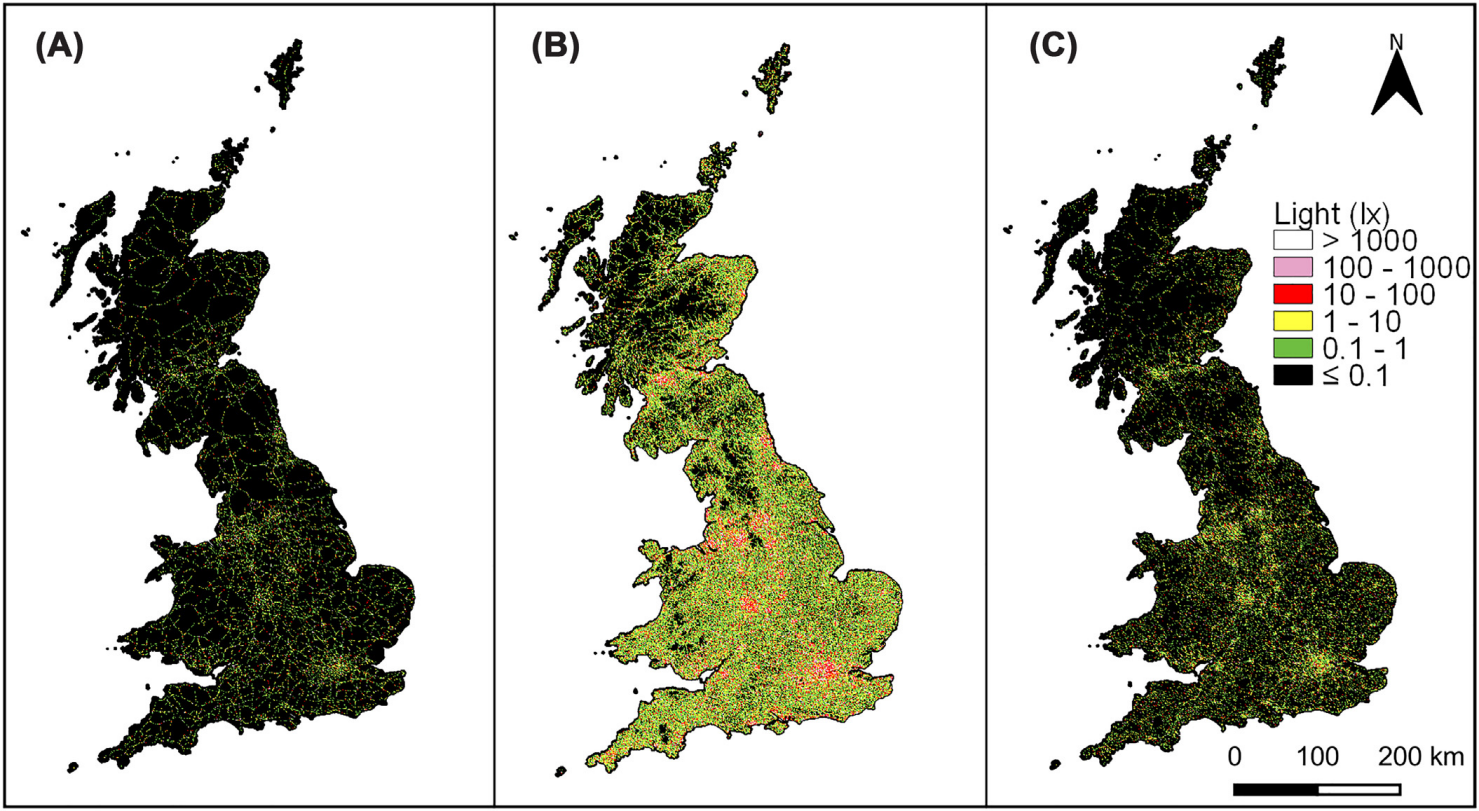
Estimated variation across Great Britain in ALAN from (**A**) streetlights and (**B**) vehicle headlights in terms of maximum exposure, that is, light level when a vehicle passes, and (**C**) average exposure, accounting for traffic volumes (based on Phillips et al. 2021). Maps (A) and (C) are modeled using a simple inverse square decay function (ignoring topography), with respect to distance from roads, and varied event frequency based on the type of roads.

Nonetheless, mobile sources may both be extensive and disproportionately contribute to ALAN beyond urban areas (where the vast majority of emissions will be from static sources, and where the majority of streetlights occur). The global road network alone is estimated to extend over 36 million km (CIA 2020), and in 2015 there were an estimated 947 million passenger cars and 335 million commercial vehicles in use (OICA 2020); in the UK, one of the only regions for which data are accessible, 16–48% of traffic is on the road outside of daylight hours, depending on the time of year (Department for Transport 2016). In many regions, road coverage is sufficiently dense that most land may be exposed to light emissions from vehicle headlights. In particular, headlights introduce artificial light into areas that do not experience streetlights, especially in rural areas (including protected areas) that may be otherwise buffered from many other anthropogenic impacts on the environment. Vehicle headlights produce a focused beam (a “bundled” source; see above) that is projected horizontally and travels further and at higher intensities than light emissions from streetlighting. For example, modeling suggests that half of land in Great Britain is less than 216 m from a road, and that around 70% of land may be exposed to vehicle headlight emissions, while only a small portion of this is exposed to emissions from streetlighting (Fig. 3; Phillips et al. 2021).

Most consideration of the environmental impacts of vehicle headlights has focused on the dazzling of vertebrates, especially mammals, and the resultant potential for causing collisions with vehicles (e.g., Outen 2002). However, particularly importantly, light emissions from vehicle headlights are commonly experienced locally as irregularly timed pulses as vehicles pass, rather than as continuous lighting. In laboratory contexts, light pulses, even when infrequent and very short lived, are capable of major perturbations to the circadian rhythms of organisms, to their visual systems and to their behavior (for review see Gaston and Holt 2018). The rapid and unpredictable fluctuations in light levels experienced by roadside organisms are likely also to lead to greater problems with physiological and behavioral habituation.

Alongside road vehicles, the other major source of mobile ALAN emissions is shipping. There were estimated to be 3.7 million marine fishing vessels in 2015 (Rousseau et al. 2019), and 98,000 vessels in the global merchant fleet in 2020 (UNCTAD 2020). These vessels can use lights for generalist (e.g., anti-collision, deck operations) or more specialist (e.g., fishing, anchor handling, diving) activities, although much is emitted simply as a consequence of a lack of routine use of blinds or other forms of blackout. Attention to the biological impacts of ALAN from shipping has focused foremost on the potential for bird strikes, particularly in regions where seabirds are abundant (Dick and Donaldson 1978; Black 2005; Merkel and Johansen 2011). However, there may be others (see below).

## Marine systems

The vast majority of research into the extent of ALAN and into its biological impacts has concerned terrestrial and freshwater systems (113/126 studies in the meta-analysis of Sanders et al. 2021). Nonetheless, ALAN is widely experienced by marine systems, and seems likely to have similarly major biological implications. Davies et al. (2014) estimated from satellite-derived data that 22% of the world’s coastlines (excluding Antarctica) were exposed to ALAN, although this is likely to be a marked underestimate for reasons already discussed. Obviously much of this coastal lighting can carry far out to sea because light paths are typically unhindered, but more locally may influence much of the extent of natural coastal ecosystems including that of, fast disappearing, tidal flats (Murray et al. 2019). Other forms of largely static ALAN emissions in marine systems arise from repeated or long-term mooring of vessels in the same localities, from offshore oil and gas platforms, and increasingly from wind turbine arrays. Shipping as a source of mobile emissions has already been mentioned, with the global coverage of its routes over even a single year being extremely widespread (e.g., Halpern et al. 2008). However, particularly significant is the use of lights, for deck operations, or explicitly as lures, by fishing vessels, with studies having demonstrated large aggregations of such lighting in some parts of the oceans (e.g., https://earthobservatory.nasa.gov/features/Malvinas).

The biological impacts of ALAN in marine systems seem likely to be at least as diverse as those occurring terrestrially, with evidence already for effects on timing of coral spawning (Ayalon et al. 2020); invertebrate settlement (Davies et al. 2015); behavior of pelagic organisms (Berge et al. 2020); turtle nesting and orientation (Thums et al. 2016; Silva et al. 2017; Hu et al. 2018; Vandersteen et al. 2020); and seabird grounding and mortality (Wiese et al. 2001; Le Corre et al. 2002; Jones and Francis 2003; Rodríguez et al. 2014; Syposz et al. 2018). Impacts on diel vertical migration of zooplankton could be especially profound, given its importance for global carbon cycling.

## Remote sources

**Fig. 4:**
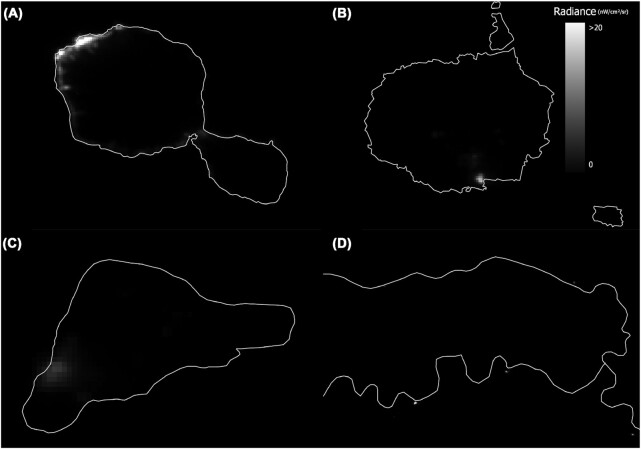
Four examples of ALAN found in remote places in the world. (**A**) island of Tahiti, a popular tourist destination in the Pacific Ocean, with most ALAN arising from the coastline because the island interior is mostly uninhabited (17.6509°S, 149.4260°W); (**B**) central island of Santa Cruz in the Galapagos archipelago, home to the Galapagos National Park, with the principal source of ALAN coming from the town of Puerto Ayora (0.6144°S, 90.3451°W); (**C**) remote volcanic archaeological site, Easter Island, with much of the island being protected as a world heritage site and the ALAN being produced by the main town, Hanga Roa (27.1127°S, 109.3497°W); and (**D**) small villages producing ALAN on the track of the Amazon river (bottom track) in South America and the Japura River (top track), while being surrounded by protected and conserved land (2°14′52.5″S 66°39′19.6″W). ALAN data from VIIRS 2019 composite.

While not distinct from the considerations mentioned thus far, the sometimes remote occurrence of ALAN emissions is worth emphasis. Such emissions occur at remote industrial sites (e.g., mines, sawmills, oil rigs), on oceanic islands, at isolated desert and forest settlements, tourist lodges and research sites, and at Arctic and Antarctic bases (Fig. 4). In the context of some other environmental pressures (e.g., plastic pollution), such occurrences often attract much scientific and media attention. Ironically, of course, demonstrating those occurrences for other pressures often itself entails the introduction—if only temporarily—of ALAN.

The impacts of its introduction, even temporarily, into “light-naïve” areas that have previously not experienced ALAN may be much greater than those in regions where ALAN has been present within the wider landscape for some time. This is suggested by historical accounts where gas or electric light was introduced for the first time. In the early 19th Century, the entomologist Doubleday (1837), for example, reported trapping moths in rural New York State by removing the glass window of a room lit by a single lamp—he cited reports that in summer hundreds of moths would be swept from the floor of the room in the morning. Early reports from lighthouses reported mass mortality of migrating birds through attraction followed by collision and/or exhaustion—10,000 birds were reported killed in one season at Gatteville lighthouse in France, and 3200 in two nights at Belle-Ile (AP 1917). More recently, gas flaring on oil production facilities in the Arctic has been shown to attract large numbers of birds (Day et al. 2015).

Remote sources of ALAN may also have disproportionate biological impacts if, as seems likely, they are often bright and poorly or unshielded, resulting in emissions above the horizontal. This may occur because of perceived needs in the absence of widespread lighting sources, because of weaker regulations, or weaker enforcement of regulations.

## Light levels

Implicit to much discussion about its biological impacts is that these will be increased by greater levels of ALAN. This may indeed often be the case, with the suppression of melatonin production, for example, showing a clear positive dose response relationship (Grubisic et al. 2019). Given that higher levels of ALAN tend often (although far from exclusively) to be associated with urban areas, this would encourage belief that these are where the biological impacts are likely to be most severe (subject to adaptation and habituation). However, a recent meta-analysis, addressing a wide variety of impacts, found no evidence for a systematic increase in effect sizes with levels of ALAN (Sanders et al. 2021). Moreover, it has been shown that the complexities of cascading effects through food webs can mean that lower intensities have greater impacts than higher ones (Sanders et al. 2018). If these results generalize, then the fact that ALAN is considerably more widely spatially distributed at lower levels than at higher ones becomes much more significant in interpreting the extent of its impacts.

## Skyglow

Thus far we have focused principally on the occurrence and biological impact of direct emissions of ALAN. In terms of the extent of ALAN then skyglow is also an important consideration. As well as being directly associated with urban areas it has been found to be detectable over distances of hundreds of kilometers from these sources (Luginbuhl et al. 2014), with its reach being greater, through amplification, on cloudy nights (Kyba et al. 2011; Jechow et al. 2017). This means that skyglow extends into many areas identified as important for biodiversity and into many areas protected for biological conservation (Garrett et al. 2020).

It is widely presumed that skyglow has biological impacts, both by influencing levels of organismal activity and by obscuring natural diel, lunar and seasonal cycles. This is reinforced by the aforementioned evidence for impacts of ALAN at low intensities. This said, studies of these biological impacts, which can have significant practical challenges, have thus far been scarce, and limited to experimental demonstrations of the influence of skyglow on the movements of organisms in aquatic systems (Moore et al. 2000; Torres et al. 2020).

Skyglow tends predominantly to be measured and modeled at the zenith, but is at its brightest on the horizon (see e.g., Jechow et al. 2017). This could serve to extend its spatial influence much further, if animals used the increased brightness of horizons either to improve their detection of prey or of predators, or for orientation (see Limpus and Kamrowski 2013). It would be surprising if some species at least did not exploit these opportunities, although it seems likely to be hard to demonstrate in the field.

## Elevation

The biological impacts of ALAN tend foremost to be thought of, and measured (including for skyglow), in terms of the emissions that are likely to be sensed or experienced by organisms at ground level. However, flying animals may (literally) have very different perspectives, and be influenced by ALAN on very different spatial scales. Ignoring atmospheric refraction and any intervening obstacles (vegetation, buildings, topography),

$$\begin{equation*}
d \approx {\rm{ }}3.57\surd h
\end{equation*}$$

where *d* is distance to the horizon (in km) and *h* is height above sea level (in m).

For example, while nocturnally migrating birds commonly fly at relatively low altitudes (a few hundred meters), they may regularly do so up to 6000–7000 m above sea level, and sometimes even higher (Liechti and Schaller 1999; Bruderer et al. 2018; Sjöberg et al. 2018). This equates to direct emissions of ALAN potentially being visible over distances of 10s to 100s of kilometers. There is evidence that this can result in these birds being attracted to ALAN sources from long distances, which may result in course adjustments through to landfall and stopover in lit areas (Bowlin et al. 2015; La Sorte et al. 2017; McLaren et al. 2018; but see Cabrera-Cruz et al. 2020).

## Landscape ecology

Aside from consideration of possible influences on migratory movements, most observations and studies of the biological impacts of ALAN have concerned local scales. This ignores the fact that ALAN could have landscape scale effects, and the extent to which ALAN influences the perceived connectivity or fragmentation of habitats, and can act as a barrier or as a facilitator of movements. By modifying or restricting patterns of movement, dispersal and migration at a landscape scale, there is potential for ALAN to have population-level effects even in landscapes where only a small proportion of the area is exposed to ALAN and for species where the duration of direct exposure (as a proportion of the life of an individual) is relatively brief. It is well known that road networks can have profound effects on the movement of animals and thus on the viability of populations (van der Ree et al. 2015), despite the actual area of land directly affected by roads being much smaller than that unaffected. Similarly, networks and patches of light may have landscape effects as barriers and population sinks. Modifying the ability of species to move through landscapes could alter metapopulation dynamics, gene flow between populations and foraging opportunities for individuals. Modeling and some field studies have shown that these effects could be very significant (e.g., Beier 1995; Bennie et al. 2014b; Laforge et al. 2019).

## Interactions with other factors

In common with most discussion of the biological impacts of ALAN, thus far we have treated this as an environmental pressure in isolation from others. However, virtually no such pressure acts entirely independently. Key therefore in understanding the biological impacts of ALAN is to consider whether it is likely to exacerbate other forms of pollution, particularly those that act unequally across the diel cycle and for which there is evidence that the impacts could be greater at night, such as global warming asymmetry (Cox et al. 2020b) and ocean acidification (Price et al. 2012).

## Future changes

We have focused throughout this piece on the current situation. However, just as important is how ALAN, and its biological impacts, will change in the future. In terms of raw infrastructure, studies have variously projected that globally urban areas (the fastest growing land use) will increase by a factor of 1.8–5.9 by 2100 (Gao and O’Neill 2020), the global road network by 1.65, and road vehicle numbers by 2.0 by 2050 (Dulac 2013). Furthermore, the vast majority of this growth (e.g., 90% of growth in road use) will come from regions outside of Europe and North America (Dulac 2013; Gao and O’Neill 2020), where existing ALAN is lower, so such growth is likely to result in a disproportionate increase in the extent of ALAN. This will serve directly to exacerbate global biological impacts of ALAN, including through many of the mechanisms highlighted in this paper.

It also seems likely that global biodiversity will continue to decline, with suggestions that even substantive steps are unlikely quickly to reverse this trend (e.g., Leclère et al. 2020). This will mean that any negative biological impacts of ALAN are likely to become proportionally yet more significant.

These effects could potentially be somewhat offset if there were strong evolutionary responses to the biological impacts of ALAN. As yet, evidence of such responses is rather limited (but see Altermatt and Ebert 2016). This might be anticipated given that the reliance on natural light cues for the timings of many components of biological activity is evolutionarily deep-rooted. However, the selection pressure exerted by ALAN may be strong (particularly where it leads to large effects on organismal fitness through reduced reproduction or increased mortality), and thus nonetheless encourage evolutionary responses. Whether the paucity of evidence for these responses is simply a consequence of a paucity of studies remains an open question.

## In conclusion

There are regions of the earth that continue to experience natural light regimes. And there are more extensive areas over which the changes to those regimes caused by ALAN are, while detectable, unlikely to have substantive biological impacts. But it is also clear that given the diversity of sources and ways in which ALAN is emitted, and the diversity of ways in which it impacts biological systems, the proportion of the earth over which ALAN occurs at levels at which it is likely to have such impacts is marked. It is certainly vastly greater than the areal coverage by cities and towns in which context it is usually discussed and more comparable to that over which many other key anthropogenic environmental pressures have impacts. Particularly given the rapidity with which the global extent of ALAN is growing, this highlights the importance of studying its biological impacts in non-urban settings and for the purpose of understanding its impacts in those settings.

## Data Availability

No new data are used in this manuscript.

## Notes

From the symposium “Blinded by the light: Effects of light pollution across diverse natural systems” presented at the annual meeting of the Society for Integrative and Comparative Biology virtual annual meeting, January 3–February 28, 2021.
